# Human papillomavirus-associated increase in p16^INK4A^ expression in penile lichen sclerosus and squamous cell carcinoma

**DOI:** 10.1111/j.1365-2133.2007.08305.x

**Published:** 2008-02

**Authors:** DM Prowse, EN Ktori, D Chandrasekaran, A Prapa, S Baithun

**Affiliations:** Centre for Molecular Oncology, Institute of Cancer John Vane Science Centre, Charterhouse Square, London EC1M 6BQ, U.K; * Pathology Group, Institute of Cell and Molecular Science, Bart’s and The London Queen Mary’s School of Medicine and Dentistry John Vane Science Centre, Charterhouse Square, London EC1M 6BQ, U.K

**Keywords:** human papillomavirus, Ki67, lichen sclerosus, p16^INK4A^, penile squamous cell carcinoma

## Abstract

**Background:**

Human papillomaviruses (HPVs) are sexually transmitted human carcinogens that may play a role in the oncogenesis of penile cancer.

**Objectives:**

To investigate the role of HPV infection and expression of the tumour suppressor protein p16^INK4A^ in the pathogenesis of penile cancer.

**Methods:**

By means of polymerase chain reaction amplification and reverse hybridization line probe assay to detect HPV infection, and immunohistochemical staining for p16^INK4A^ and Ki67, we analysed 26 penile squamous cell carcinomas (SCCs) and 20 independent penile lichen sclerosus (LS) lesions from 46 patients.

**Results:**

HPV DNA was found in 54% of penile SCCs and 33% of penile LS cases in single and multiple infections. High-risk HPV 16 was the predominant HPV type detected. No relationship between Ki67 expression and HPV infection was observed. Strong immunostaining for p16^INK4A^ correlated with HPV 16/18 infection in both penile LS and penile SCC. In our penile SCC series the cancer margins were also associated with penile LS in 13 of 26 lesions, and HPV was detected in seven of the 13 SCC cases associated with LS and in six of the 11 SCC lesions not involving LS.

**Conclusions:**

Our study shows a high prevalence of HPV 16 and p16^INK4A^ expression in penile lesions, consistent with an active role for HPV in interfering with the retinoblastoma pathway. High-risk HPV infection could be involved in the tumorigenic process in 50% of penile cancers, and the use of prophylactic HPV vaccines has the potential to prevent these cancers.

Penile cancer accounts for 0·3–0·6% of all cancers in men in the Western world and this incidence is much higher in some African, Asian and South American countries.^1^,^2^ Early diagnosis of penile cancer is both lifesaving and essential for cosmetically acceptable treatment. The most common histological type of penile cancer is squamous cell carcinoma (SCC), accounting for 95% of cases. Its aetiology is unknown, but risk factors include age and lack of circumcision.^1^,^2^ Penile lichen sclerosus (LS) or balanitis xerotica obliterans is a chronic inflammatory disorder of unknown cause that may be a predisposing factor to the development of penile SCC, as 3–6% of patients with penile LS are reported to develop penile carcinoma^3^,^4^ and 28–50% of penile SCC cases have associated LS.^5^–^7^ Vulval LS is also considered one of two pathways for the development of vulval cancer, the other being via human papillomavirus (HPV) infection.^8^

Persistent infection with sexually transmitted high-risk HPV is the main cause of cervical cancer,^9^,^10^ and HPV infection may also play a role in penile carcinogenesis.^11^ A common aetiology for penile and cervical cancer is suggested by the geographical correlation between the incidence of penile and cervical cancers worldwide.^12^ Male circumcision, which is associated with a reduced risk of penile cancer,^1^,^2^ has also been associated with decreased penile HPV infection and, in the case of men with a history of multiple sexual partners, a reduced risk of cervical cancer in their current female partners.^13^ The prevalence of HPV penile infections in healthy men is 3–9% in Western Europe which has a low incidence of penile cancer and 39% in Brazil where penile cancer rates are higher.^14^,^15^ However, unlike cervical cancer where it is considered that all cancers are HPV related and HPV infection can be detected in almost all cases,^9^ the infection rates of HPV in penile cancer range from 20% to 80%, depending on detection method and geographical location.^1^,^2^

HPV contributes to tumorigenesis predominantly through the action of the viral oncoproteins (E6 and E7). The interaction of E7 with the retinoblastoma (Rb) tumour suppressor leads to Rb degradation, E2F activation and overexpression of the cyclin-dependent kinase inhibitor p16^INK4A^.^16^,^17^ The objective of this study was to examine the prevalence of HPV DNA in penile LS and SCC and to investigate whether penile HPV infection is associated with p16^INK4A^ expression in both types of lesion.

## Materials and methods

### Patient samples, DNA preparation and human papillomavirus genotyping

Archival paraffin wax-embedded tissue sections from 26 penile SCCs, 20 penile LS cases not associated with SCC and 26 cervical cancers (Barts and The London NHS Trust) were obtained and reviewed by a pathologist (S.B.), with ethical approval from the East London and City Health Authority Research Ethics Committee (P-02-84 and P-03-302). DNA was extracted from paraffin wax-embedded sections using the QIAamp DNA Mini kit (Cat. No. 51304; Qiagen, Crawley, U.K.). β-Globin polymerase chain reaction (PCR) was performed using primers B1 and B19 to confirm the adequacy of the extracted DNA. Validated samples were tested for the presence of HPV DNA by a broad-spectrum HPV PCR method using SPF10 primers which amplify a 65-bp fragment of the L1 open reading frame.^18^ HPV genotypes were identified by the INNO-LiPA line probe assay (Innogenetics NV, Ghent, Belgium).^19^,^20^

### Immunohistochemistry

Antigen retrieval using citrate buffer was performed on dewaxed sections prior to blocking with swine serum. Primary antibodies to Ki67 (MIB-1; Dako Cytomation, Glostrup, Denmark; M 7240, dilution 1 : 50) and p16^INK4A^ (Ab-7; Neomarkers, Fremont, CA, U.S.A.; MS-1064-P0, dilution 1 : 100) were applied for 1 h and detected using an Envision Kit (Dako Cytomation; K4006). p16^INK4A^ staining was assessed by grading the intensity (0, negative; 1, low intensity; 2, mid range; 3, high intensity) and percentage of positively stained cells (1, low percentage coverage, 0–33%; 2, mid percentage coverage, 34–67%; 3, high percentage coverage, 68–100%). The combined score total was calculated by adding together the intensity and percentage coverage scores. Ki67 staining was assessed by the percentage of positively stained cells only. Statistical analysis was performed using Fisher’s exact test and SPSS software (SPSS, Chicago, IL, U.S.A.). *P* < 0·05 was considered significant.

## Results

An analysis of tissue samples from 26 penile carcinomas, 20 penile LS cases not associated with SCC and 26 cervical cancers was performed to determine HPV prevalence and genotype (Table 1). Two cases of penile LS were excluded from study due to negative β-globin results. High-risk HPV infection was present in 14 of 26 (54%) penile cancers, six of 18 (33%) penile LS cases and 24 of 26 (92%) cervical cancers (Table 1). HPV infections were significantly lower in penile LS and SCC than in cervical carcinoma (*P* < 0·05). There was no significant difference in the number of multiple HPV infections between penile LS and SCC. For both penile LS and SCC, HPV-positive cases consisted of 50% single infections and 50% multiple infections. Similar frequencies were observed in our cervical cancer series (58% single infections and 42% multiple infections). High-risk HPV genotypes were detected in all HPV-positive penile lesions and cervical cancers. Only high-risk HPV 16 and 18 were found as single infections in penile cancer. HPV 16, which is strongly associated with malignant mucosal lesions, was the most commonly occurring HPV type in single and multiple HPV infections in penile LS, penile SCC and cervical cancers. HPV 16 was detected in all six HPV-positive penile LS cases, 11 of 14 (79%) HPV-positive penile SCCs and 20 of 22 (91%) HPV-positive cervical cancers. High-risk HPV 18, 39, 45, 52 or 68 infections were detected in the three HPV-positive penile cancers lacking HPV 16 and in some of the multiple infections with HPV 16 and HPV 6, 33, 40, 42, 51 or 56.

**Table 1:** Human papillomavirus (HPV) DNA polymerase chain reaction and genotype distribution in penile lichen sclerosus (LS), penile squamous cell carcinoma (SCC) and cervical carcinoma

			HPV genotype distribution																	
Diagnosis	*n*	HPV	16	18	53	16	16	16	16	16	40^a^	16	16	16	18	11^a^	16	39	16	6^a^
		DNA				18	33	56	52	66	52	52	56	33	45	16	45	68	18	16
		Positive										68	18	56	68	56	56	52	33	42^a^
																			51	44^a^
Penile LS	18	6 (33%)	3	0	0	1	1	0	0	0	0	0	0	0	0	0	0	0	1	0
Penile SCC	26	14 (54%)	6	1	0	0	0	3	0	0	1	1	0	0	0	0	0	1	0	1
Cervical carcinoma	26	24 (92%)	13	0	1	1	0	2	1	1	0	0	1	1	1	1	1	0	0	0

a Low risk or unclassified HPV genotype.

**Fig 1:**
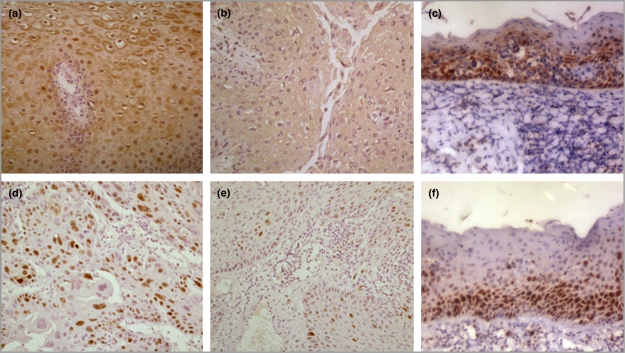
Representative examples of p16^INK4A^ and Ki67 immunohistochemistry showing (a) high levels of p16^INK4A^-immunoreactive penile squamous cell carcinoma (SSC) [note positive nuclear and cytoplasmic staining (brown)]; (b) low levels of p16^INK4A^-immunoreactive penile SSC; (c) high levels of p16^INK4A^-immunoreactive penile lichen sclerosus (LS); (d) high levels of Ki67-immunoreactive penile SSC [note positive nuclear staining (brown)]; (e) low levels of Ki67-immunoreactive penile SSC; and (f) high levels of Ki67-immunoreactive penile LS.

We examined the expression of proliferation marker Ki67 and p16^INK4A^ in penile LS and SCC by immunohistochemistry. Staining for Ki67 and p16^INK4A^ was observed in both penile LS and SCC (Fig. 1). No relationship between Ki67 expression and p16^INK4A^ or HPV infection was observed (Table 2). In contrast, strong immunostaining for p16^INK4A^ was found in approximately 50% of the penile lesions and this was associated with HPV 16/18 infection in both penile SCC and penile LS (*P* < 0·05) (Table 2).

**Table 2:** p16^INK4A^ and Ki67 expression in penile lichen sclerosus (LS), penile squamous cell carcinoma (SCC) and cervical carcinoma

		p16^INK4A^		Ki67	
Diagnosis	HPV PCR	0–3	4–6	0–1	2–3
Penile LS	HPV 16/18+	0	6*	0	6
	HPV−	8	4	0	12
	Total	8(45%)	10(55%)	0(0%)	18(100%)
Penile SCC	HPV 16/18+	4	8*	4	8
	HPV other+	2	0	0	2
	HPV−	9	3	3	9
	Total	15(54%)	11(46%)	7(27%)	19(73%)
Cervicalcarcinoma	HPV 16/18+	3	20	11	12
	HPV other+	0	1	0	1
	HPV−	0	2	0	2
	Total	3(12%)	23(88%)	11(42%)	15(58%)

PCR, polymerase chain reaction.

* In penile LS and SCC high levels of expression of p16^INK4A^ are significantly associated with high-risk human papillomavirus (HPV) 16/18 infection (*P* < 0·05).

In our penile SCC series the cancer margins were also associated with penile LS in 13 of 24 cases (54%), with two cases uncharacterized due to insufficient noncancerous material. HPV was detected in seven of the 13 (54%) SCC cases associated with LS and in six of the 11 (55%) SCC cases not involving LS.

## Discussion

We have established the prevalence of HPV DNA in penile LS and SCC and found an association with high-risk HPV 16/18 and p16^INK4A^ expression. Penile LS has been proposed to be a premalignant lesion predisposing for penile cancer development.^5^,^6^ However, there is only one report of the incidence of HPV infection in penile LS. Our detection of high-risk HPV 16 in 33% of our penile LS cases is higher than in a recent Italian study, which found it in 17% of penile LS cases. We detected HPV infections in 54% of our penile cancer cases, which is consistent with recent reports that have found HPV in 30% of Dutch^21^,^22^ and 77% of Spanish^23^ penile carcinomas. HPV infections were more common in our penile cancers (54%) than in LS cases (33%). We found concurrent infection with more than one HPV type in 50% of infected subjects in both penile LS and SCC. Multiple infections have not previously been reported in penile LS^15^ but have been observed in up to 20% of penile SCCs.^22^,^24^,^25^ Multiple HPV infections have also been reported in patients with cervical abnormalities or cancers^26^ and in some studies but not others this has been associated with a higher risk of cervical intraepithelial neoplasia.^27^,^28^ However, in HPV co-infections one HPV type may proliferate causing pathogenesis while the others are latent bystanders.^26^

High-risk HPV 16 was the most prevalent HPV genotype detected in each of our penile series, infecting 100% of HPV-positive penile LS and 79% of HPV-positive penile SCC cases. HPV 16 and HPV 18 were the only HPV genotypes occurring as single infections, suggesting that these genotypes are more likely to contribute to the carcinogenic process. HPV 16 and 18 are the predominant high-risk HPV genotypes associated with cervical cancer.^12^,^26^ HPV 16 was the most frequently detected genotype in Italian patients with LS, found in six of eight HPV-positive cases.^15^ Our study of HPV infection in penile carcinoma is in accordance with most other investigations which find HPV 16 as the commonest genotype detected in Europe,^21^–^23^ although in Argentina and Thailand HPV 18 can predominate.^29^,^30^

p16^INK4A^ expression in penile LS has not previously been reported, and we are aware of only one paper reporting p16^INK4A^ expression in penile cancer.^21^ The expression of p16^INK4A^ and the proliferation marker Ki67 has been described in gynaecological pathology.^16^,^17^,^31^ We found that expression of Ki67 was high in all cases of penile LS and 73% of penile SCCs and did not correlate with HPV infection. In contrast, strong expression of p16^INK4A^ which occurred in 55% of penile LS and 46% of penile SCC cases was significantly associated with HPV 16/18 infection. This association is in agreement with Ferreux *et al.* who reported that increased p16^INK4A^ expression correlated with HPV detection and occurred in 29% of penile SCCs,^21^ although we observed higher levels of HPV infection and p16^INK4A^ expression in our series. In the two cervical cancer, three penile SCC and four penile LS cases where p16^INK4A^ was strongly expressed and no HPV was detected, the levels of HPV may be too low for detection or HPV types may be present that are not amplified by the SPF10 system.^18^ In the absence of HPV infection p16^INK4A^ expression is frequently inhibited by methylation or mutation in penile SCC^21^,^32^ and nonpenile cutaneous SCC.^33^ Inactivation of p16^INK4A^ indirectly by HPV or by methylation and mutation therefore appears to be an important step in penile tumorigenesis.

Our observation that penile SCC was associated with LS in 54% of our cancer cases is consistent with LS being a predisposing factor to the development of penile and vulval SCC.^5^–^8^ However, vulval SCC linked to LS has not been associated with HPV infection and conflicting results have been reported for the involvement of HPV in the development of SCC in patients with penile LS. Perceau *et al.* found HPV in five of nine SCCs without LS association and only one of eight SCCs with LS association,^34^ while in contrast Nasca *et al.* reported HPV 16 infection in two of three penile SCCs that developed in a series of 86 patients with LS.^3^ Our results show high-risk HPV 16 infections to occur in patients with LS. We also found HPV 16 infections at a similar frequency (50%) in penile SCC with LS association and in penile SCC without LS association. Persistent HPV infection could be involved in the tumorigenic process in approximately 50% of penile cancers, as unresolved HPV infection is a risk factor for cervical cancer.^20^

The importance of HPV status in penile cancer progression and patient survival is controversial. High-risk HPV has traditionally been associated with aggressive variants,^35^ but recent series examining the relationship of HPV infection with prognosis have revealed either no correlation with lymph node metastasis and survival^25^ or a favourable survival outcome.^22^

In summary, high-risk HPV infection occurs in both penile LS and SCC and is associated with p16^INK4A^ expression. Expression of p16^INK4A^ may therefore be of value as a surrogate marker confirming the diagnosis of HPV-infected premalignant and malignant penile lesions. HPV 16 predominates in penile LS and SCC and it is likely to be an aetiological agent in the development of a significant proportion of penile cancers. These results are important as prophylactic HPV vaccines being developed for prevention of cervical cancer in women could also prevent penile cancers in men.
